# Multi-Target Approaches in Metabolic Syndrome

**DOI:** 10.3389/fphar.2020.554961

**Published:** 2021-03-12

**Authors:** Felix F. Lillich, John D. Imig, Ewgenij Proschak

**Affiliations:** ^1^ Institute of Pharmaceutical Chemistry, Goethe-University of Frankfurt, Frankfurt, Germany; ^2^ Department of Pharmacology and Toxicology, Medical College of Wisconsin, Milwaukee, WI, United States

**Keywords:** metabolic sydrome, polypharmacology, multi-target drug, peroxisome prolifer ators-activated receptor-γ, soluble epoxide hydrolase, farnesoid X receptor

## Abstract

Metabolic syndrome (MetS) is a highly prevalent disease cluster worldwide. It requires polypharmacological treatment of the single conditions including type II diabetes, hypertension, and dyslipidemia, as well as the associated comorbidities. The complex treatment regimens with various drugs lead to drug-drug interactions and inadequate patient adherence, resulting in poor management of the disease. Multi-target approaches aim at reducing the polypharmacology and improving the efficacy. This review summarizes the medicinal chemistry efforts to develop multi-target ligands for MetS. Different combinations of pharmacological targets in context of *in vivo* efficacy and future perspective for multi-target drugs in MetS are discussed.

## Introduction

**FIGURE 1:**
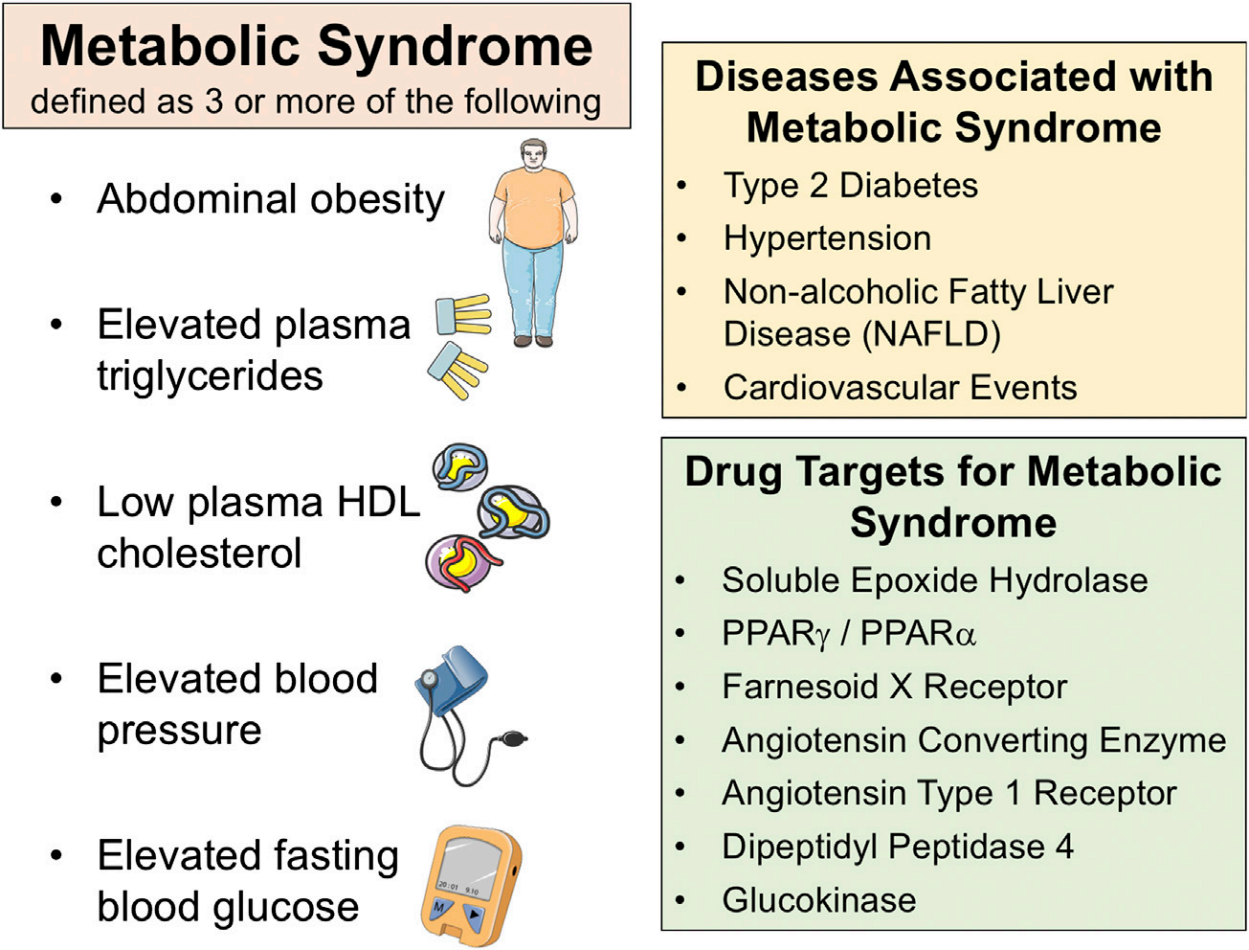
Metabolic syndrome is defined as three or more of the following; abdominal obesity, elevated plasma triglycerides, low plasma HDL cholesterol, elevated blood pressure, elevated fasting blood glucose (**left panel**). There are several diseases associated with metabolic syndrome (**right top panel**). Several drug targets have been identified for metabolic syndrome and associated diseases (**right bottom panel**).

Metabolic diseases are becoming increasingly prevalent and have a major impact on public health worldwide (Kahn et al., 2005; Potenza and Mechanick, 2009; Shaw et al., 2010). Major metabolic diseases include metabolic syndrome (MetS), type 2 diabetes, and non-alcoholic fatty liver disease (NAFLD). Insulin resistance is a key component for MetS and metabolic diseases such as type 2 diabetes and NAFLD. MetS patients are diagnosed when they have at least three of the following risk factors: abdominal obesity, hypertriglyceridemia, elevated blood pressure, low high-density lipoprotein (HDL) cholesterol, or glucose intolerance Figure 1 (Grundy et al., 2004; Alberti et al., 2005; Grundy, 2006). Type 2 diabetes afflicts close to 200 million worldwide and type 2 diabetic patients have obesity-related MetS (Grundy et al., 2004; Potenza and Mechanick, 2009; Shaw et al., 2010). Diabetes is already the leading cause of blindness, end-stage liver disease, and end-stage renal disease (Alberti et al., 2005; Kahn et al., 2005). Likewise, NAFLD is the most common chronic liver disease and affects up to one-third of the adult population (Basaranoglu and Neuschwander-Tetri, 2006; Sumida and Yoneda, 2018). A large portion of patients with NAFLD display typical features of MetS including abdominal obesity, dyslipidemia, hypertension, insulin resistance, or type 2 diabetes (Chalasani et al., 2012; El-Kader and El-; El-Kader and El-Den Ashmawy, 2015). Developing adequate therapeutic and preventive measures for these multifactorial metabolic diseases has so far been challenging. Indeed, due to the complex pathophysiology, the current therapeutic approaches to treat MetS, type 2 diabetes, and NAFLD need multiple treatments regulating lipid and glucose homeostasis as well as blood pressure control (Grundy et al., 2005; Grundy, 2006; Oseini and Sanyal, 2017; Sumida and Yoneda, 2018).

Apart from the metabolic abnormalities per se, inflammation associated with these metabolic diseases plays a crucial role in increasing cardiovascular events, causing NAFLD progression to hepatocellular cancer, and causing diabetic complications such as nephropathy, neuropathy, and retinopathy (Dandona et al., 2005; Esser et al., 2014). Metabolic diseases induce disruption in many physiological regulatory systems due to excessive energy intake that provokes stressor stimuli that subsequently trigger inflammatory pathways (Dandona et al., 2005; Hotamisligil, 2006; Esser et al., 2014). Increases in plasma free fatty acids also plays a critical role in the pathogenesis of insulin resistance and inflammation in metabolic diseases (Alberti et al., 2005; Forbes and Cooper, 2013). Indeed, chronic inflammation is a hallmark of metabolic disease and its severity depends on the presence of different metabolic pathophysiological components (Dandona et al., 2005; Hotamisligil, 2006; Esser et al., 2014). Humans with metabolic diseases have increased plasma concentrations of tumor necrosis factor-α (TNF-α), interleukin-6 (IL-6), IL-1β, C-reactive protein and other inflammatory mediators (Dandona et al., 2005; Hotamisligil, 2006; Esser et al., 2014). Inflamed tissues in metabolic diseases include adipose, liver, kidney, and pancreas (Dandona et al., 2005; Hotamisligil, 2006; Esser et al., 2014). Experimental studies in metabolic disease animal models demonstrate substantial macrophage infiltration and increased cytokines in abdominal and peri-renal fat tissue, which could serve as a means for inflammatory cytokines to cause cardiovascular, neural, renal, and liver complications (Dandona et al., 2005; Esser et al., 2014; Bernardi et al., 2018). These findings support the notion that therapeutics that have anti-inflammatory actions could broadly combat metabolic diseases and associated complications.

Current therapeutics for metabolic diseases are varied in terms of ability to improve patient outcomes (Grundy et al., 2005; Grundy, 2006; Oseini and Sanyal, 2017). In addition, there are currently no approved drugs for the treatment of NAFLD (Oseini and Sanyal, 2017; Sumida and Yoneda, 2018). A first-line therapeutic approach for metabolic diseases is lifestyle changes including diet modification and physical activity (Grundy et al., 2005; Grundy, 2006). Nonetheless, often these lifestyle modifications are insufficient to normalize risk factors in patients, and treatment requires multi-drug therapy. A multi-drug regimen or polypharmacy is a major problem for the treatment of patients with metabolic diseases due to poor patient compliance, side effects, and drug-drug interactions (Grundy, 2006; Noale et al., 2016; Alwhaibi et al., 2018).

Polypharmacy for metabolic diseases includes lipid lowering agents, anti-hypertensive agents, anti-diabetic agents, heart failure drugs, and anti-obesity therapies. Reduction of low-density lipoprotein (LDL) cholesterol by statins is a common treatment for patients with metabolic diseases. Statins are 3-hydroxy-3-methyl-glutaryl-coenzyme A (HMG-CoA) reductase inhibitors that effect the rate limiting step in cholesterol biosynthesis (Grundy, 2006; Bianchi et al., 2007). Another lipid lowering approach in metabolic diseases is to inhibit intestinal cholesterol absorption with ezetimibe (Grundy, 2006; Bianchi et al., 2007). Angiotensin system inhibitors are commonly used to combat hypertension associated with metabolic diseases (Grundy et al., 2005; Bianchi et al., 2007). Angiotensin converting enzyme inhibitors or angiotensin receptor blockers not only lower blood pressure but can decrease heart and kidney disease progression in diabetes (Bianchi et al., 2007). Heart failure in metabolic disease is treated with diuretics, beta-adrenergic blockers, or calcium channel blockers (Dandona et al., 2005; Bianchi et al., 2007). Anti-diabetic agents include peroxisome proliferator-activated receptor-γ (PPARγ) agonist insulin sensitizing agents, glucagon-like peptide-1 (GLP-1) receptor agonists, dipeptidyl peptidase-4 (DPP4) inhibitors, and sodium-glucose cotransporter-2 (SGLT-2) inhibitors (Bianchi et al., 2007). These anti-diabetic agents are effective in lowering blood glucose and hemoglobin A1c (HbA1c) levels (Grundy, 2006; Bianchi et al., 2007). Obesity can be treated with gastrointestinal lipase inhibitors, central nervous system acting drugs like serotonin 2C receptor agonists, or by bariatric surgery (Bianchi et al., 2007). A primary therapeutic approach for metabolic diseases has been to identify and treat each symptom or condition separately. This approach leads to several drugs being prescribed to patients with metabolic diseases.

Patients with metabolic diseases have a high rate of polypharmacy and this is a growing concern in the elderly population (Grant et al., 2003; Noale et al., 2016; Alwhaibi et al., 2018). For example, when polypharmacy is defined as at least five drugs being prescribed, the rate of polypharmacy reaches levels as high as 60% in patients over 65 years old with non-insulin dependent type 2 diabetes (Noale et al., 2016; Alwhaibi et al., 2018). The majority of these diabetics are on at least one anti-diabetic drug, not less than one anti-hypertensive drug, and a lipid lowering statin drug (Grant et al., 2003; Grundy, 2006; Noale et al., 2016; Alwhaibi et al., 2018). Although compliance and drug-drug interactions are issues with polypharmacy, a major issue that leads to suboptimal adherence by patients are side effects associated with one or more drugs (Grant et al., 2003). PPARγ agonists are an example of an effective anti-diabetic drug with a side effect that decreased compliance and prescriptions. Edema associated with thiazolidinedione (TZD) PPARγ agonist use led to a problem with patient compliance and a warning label for use in heart failure (Guan et al., 2005). Consequently, these side-effects resulted in a drastic reduction in the use of PPARγ agonists in the treatment of type 2 diabetes (Stafylas et al., 2009). Another significant issue is the utilization of central nervous system anti-obesity drugs and side effects associated with these drugs. This concern came to light with the introduction of the cannabinoid type 1 (CB1) receptor antagonist rimonabant in 2006 (Christensen et al., 2007; Després et al., 2008). Rimonabant was effective in reducing appetite through inhibition of central nervous system CB1 receptors combined with decreasing blood glucose as well as fat and lipid metabolism via blocking peripheral CB1 receptors (Christensen et al., 2007; Després et al., 2008). Unfortunately, major side effects for rimonabant were depression and suicide that led to it being withdrawn in 2008 (Christensen et al., 2007; Després et al., 2008). Taken as a whole, metabolic diseases are treated with anti-obesity, anti-diabetic, lipid lowering, and anti-hypertensive drugs that have central nervous system and peripheral actions that lead to significant polypharmacy concerns.

A potential solution to polypharmacy in metabolic diseases is combining multiple drugs or invent drugs with multiple actions to combat metabolic diseases. Consequently, there is growing interest in developing novel therapeutic strategies that could target multiple metabolic disease components to provide a comprehensive treatment approach (Rollason and Vogt, 2003; Morphy and Rankovic, 2005). Developing a drug that lowers plasma lipids, decreases inflammation, decreases blood pressure, and lowers blood glucose levels would be ideal to treat metabolic diseases. Currently, there are no approved drugs that can reliably reduce multiple conditions associated with metabolic diseases over the long-term (Grundy et al., 2005; Grundy, 2006; Handelsman and Jellinger, 2011).

Interestingly, there are several metabolic disease drugs that have actions at multiple targets or have multiple therapeutic actions that could serve as a starting point for developing novel bifunctional molecules to treat metabolic diseases. These drugs include statins, metformin, PPAR agonists, and farnesoid X receptor (FXR) agonists. Statins have a primary action to lower LDL-c levels in metabolic diseases (Dandona et al., 2005; Grundy et al., 2005). These lipid lowering actions are due to statins ability to inhibit HMG-CoA reductase (Dandona et al., 2005; Grundy et al., 2005). Atorvastatin in particular has also been demonstrated to enhance hepatic insulin sensitivity and signaling through undetermined mechanisms (Dandona et al., 2005; Grundy et al., 2005). There is also evidence that statins have a direct anti-inflammatory action since statins lower C-reactive protein levels (Dandona et al., 2005). Another potential starting point is the biguanide metformin that is widely prescribed for type 2 diabetes and MetS (Dandona et al., 2005; Bianchi et al., 2007). Metformin lowers blood glucose and HbA1c levels and prevents weight gain (Bianchi et al., 2007). The primary action for metformin is to lower hepatic glucose output through adenosine monophosphate (AMP) kinase activation (Bianchi et al., 2007). Another positive aspect is that metformin decreases circulating plasminogen activator inhibitor-1 levels which could combat cardiovascular diseases associated with type 2 diabetes and MetS (Dandona et al., 2005; Bianchi et al., 2007). PPARγ agonists such as rosiglitazone are used as insulin-sensitizing agents to treat type 2 diabetes (Bianchi et al., 2007). Because PPARγ agonists act as a ligand regulated transcription factor these drugs regulate the expression of many genes that have a myriad of actions. One of the actions that these thiazolidinedione PPARγ agonists have is to decrease inflammatory genes to prevent atherosclerotic complications associated with type 2 diabetes (Bianchi et al., 2007). There have been attempts made to develop drugs that act on multiple PPARs including PPARα, PPARγ, and PPARδ for treating metabolic diseases (Dandona et al., 2005; Bianchi et al., 2007; Jain et al., 2018). Regrettably, these dual-acting PPARs have failed to become approved for treating metabolic diseases. Nevertheless, the strategy to introduce PPARγ activity has been excessively used in various approaches to multi-target drugs for MetS and were excellently reviewed by Ammazzalorso et al. (2019) Although not currently approved for humans, FXR agonists are being developed to treat metabolic diseases like NAFLD and fibrotic diseases (Ali et al., 2015; Oseini and Sanyal, 2017; Sumida and Yoneda, 2018). FXR activation reduces hepatic fat accumulation and has been demonstrated to have anti-fibrotic and anti-inflammatory actions (Oseini and Sanyal, 2017; Sumida and Yoneda, 2018). Obeticholic acid is an FXR agonist that has entered clinical trials for non-alcoholic steatohepatitis (NASH) and NAFLD; however, this FXR agonist has the unwanted effect of increasing total cholesterol levels and increasing the HDL-c to non-HDL-c ratio (Mudaliar et al., 2013; Han, 2018). Nevertheless, a common denominator for this group of metabolic disease drugs that could serve as a starting point to develop multi-target drugs is their ability to combat inflammation. Novel bifunctional molecules described in this review build on this anti-inflammatory action and will have the capacity to impact multiple factors including blood pressure, lipid and triglyceride levels, and insulin signaling in metabolic diseases like MetS, type 2 diabetes and NAFLD.

Rational approaches to design multi-target drugs have become popular in the last decade (Morphy and Rankovic, 2005). Most often, they comprise small molecule design which addresses two or more targets. As mentioned before, a multi-target drug potentially exhibits few side effects than a combination of drugs, leading to an improved adherence of the patients (Morphy and Rankovic, 2005). The first and crucial step is the identification of a suitable target combination. It seems to be apparent to choose targets modulating the different components of the MetS which are addressed by established drugs used in treatment of metabolic disorders. However, not every target combination represents a promising approach for the development of a multi-target ligand. First, the chemical tractability has to be taken in account, which is obviously high if the targets of interest accommodate chemically similar endogenous ligands. However, several exceptions exist, where targets with very different ligands can be addressed by one drug. The most prominent example are dual modulators of angiotensin II receptor subtype 1 (AT1) and PPARγ, which is discussed later. Second, a promising target combination exhibits a synergistic effect, contributing to the efficacy and safety of the multi-target drug. The systematic identification of synergistic target combinations, well-established in cancer and antibiotics, is still in its infancy in the field of metabolic diseases (Proschak et al., 2019).

The medicinal chemistry approaches to designed multi-target ligands can be classified in three types: linked, fused, and merged pharmacophores (Morphy and Rankovic, 2005; Proschak et al., 2019). Linking of two (or more) pharmacophores by a flexible linker often leads to compounds exhibiting sufficient *in vitro* potency toward all targets of interest, however the larger molecular weight combined with an increased number of free rotatable bonds have a negative effect on pharmacokinetics (Mor phy and Rankovic, 2006). Therefore, the identification of a pharmacophore element to enable the fusion of two ligands usually leads to smaller molecules, while the identification of a merged pharmacophore for both targets can be regarded as the most promising way to develop a multi-target ligand exhibiting a promising pharmacodynamics and pharmacokinetic profile.

Multi-target ligands have varying IC_50_ and EC_50_ that are determined by *in vitro* means. Yet, the ability to translate to *in vivo* systems is more complicated than single target ligands. A primary question that remains unanswered is whether multi-target ligands can engage two targets *in vivo* at the same time. The ability for multi-target ligands to engage two targets *in vivo* at the same time would depend on the type of multi-target molecule, cellular proximity of the targets, and binding properties to the targets. Linked multi-target ligands with excellent binding properties for two targets in very close cellular proximity is the most likely scenario where both targets could be engaged *in vivo* at the same time. A more likely scenario is that a certain amount of multi-target molecule engages each target separately with a particular plasma level required for proper modulation of the two targets. In the end, extensive *in vivo* evaluation is needed for assessing proper target engagement of multi-target ligands. Multi-target ligands described in this review have been used at doses in animal disease models that have been verified to achieve proper *in vivo* target engagement.

This review article aims at summarizing several examples of small molecule multi-target ligands for metabolic diseases. We particularly try to explain the rationale behind the choice of a certain target combination. Furthermore, we address the challenges which arise from the design and optimization of multi-target ligands and their potential in the treatment of MetS.

### sEH and PPARγ

The arachidonic acid (AA) cascade presents one of the major pathways for the progression of pain and inflammation signals in the human body. Therefore, the metabolites, enzymes and receptors of this cascade are excellent therapeutic targets for treatment of the MetS and inflammatory diseases in general (Imig and Hammock, 2009). Throughout the Cytochrome P450 (CYP)-branch AA gets metabolized into either pro-inflammatory hydroxyeicosatrienoic acid 20-HETE or to anti-inflammatory epoxyeicosatrienoic acids (EETs) (Spector, 2009). The positive effects of the different EETs are well described in literature (Spector and Norris, 2007; Shen and Hammock, 2012; Morisseau and Hammock, 2013; Campbell et al., 2017), but through further metabolization their beneficial characteristics are decreased. In humans, EETs are predominantly metabolized via the soluble epoxide hydrolase (sEH, EPHX2) pathway (Morisseau and Hammock, 2013). While epoxide hydrolases in general are a group of enzymes that converts epoxide containing compounds into their corresponding diols (Morisseau, 2013), the sEH in particular converts EETs into the less biological active dihydroxyepoxyeicosatrienoic acids (DHETs). Furthermore, the sEH forms a domain-swapped homodimer, which is encoded by the gene EPHX2, located on chromosome 8 (Tanaka et al., 2008). The protein is a bifunctional enzyme that exhibits two different catalytic sites. The already described hydrolase domain is linked via a proline rich linker to a phosphatase domain whose physiological role is still under investigation (Kramer and Proschak, 2017). In mammalian tissue the sEH is widely spread, including organs like liver, kidney, lungs, heart, brain, spleen (Newman et al., 2005). The distribution of the protein is tissue dependent on subcellular level, and the sEH can be found either in the cytosol or additionally in the peroxisomes (Przybyla-Zawislak et al., 2003).

As mentioned before, peroxisome proliferator-activated receptors (PPARs) are ligand-activated transcription factors of the nuclear receptor family, which belongs to the superfamily of nuclear receptors (Michalik et al., 2006). So far three isoforms are described in literature: α, β/δ and γ. They all form a heterodimer with the retionoid-X-receptor (RXR), which in turn is activated through 9-cis retinoic acid (Germain et al., 2006). PPARγ itself plays a major role in regulation of lipid metabolism, glucose homeostasis, adipogenesis, and in several inflammatory processes and was therefore described as the master transcriptional regulator for the development of adipose cells (Tontonoz and Spiegelman, 2008).

The sEH metabolites EETs and PPARγ signaling are tightly interconnected. Inhibition of sEH leads to increased EET levels, which were identified as endogenous ligands of PPARγ, have angiogenetic properties and increase vascular endothelial growth factor (VEGF) (Liu et al., 2005; Spector, 2009). Thus, Xu and Hammock hypothesized that the mechanism underlying the effect of the sEH inhibitor *t*-AUCB (**1**) (Hwang et al., 2007) on epithelial progenitor cells (EPCs) was the EETs-PPARγ pathway. This pathway was supported by the sEH-mediated angiogenetic effects, which in turn increases VEGF and Hypoxia-inducible factor 1-alpha (HIF-1α), triggers EPC migration and proliferation and in the end could lead to angiogenesis (Xu et al., 2013).

**FIGURE 2:**
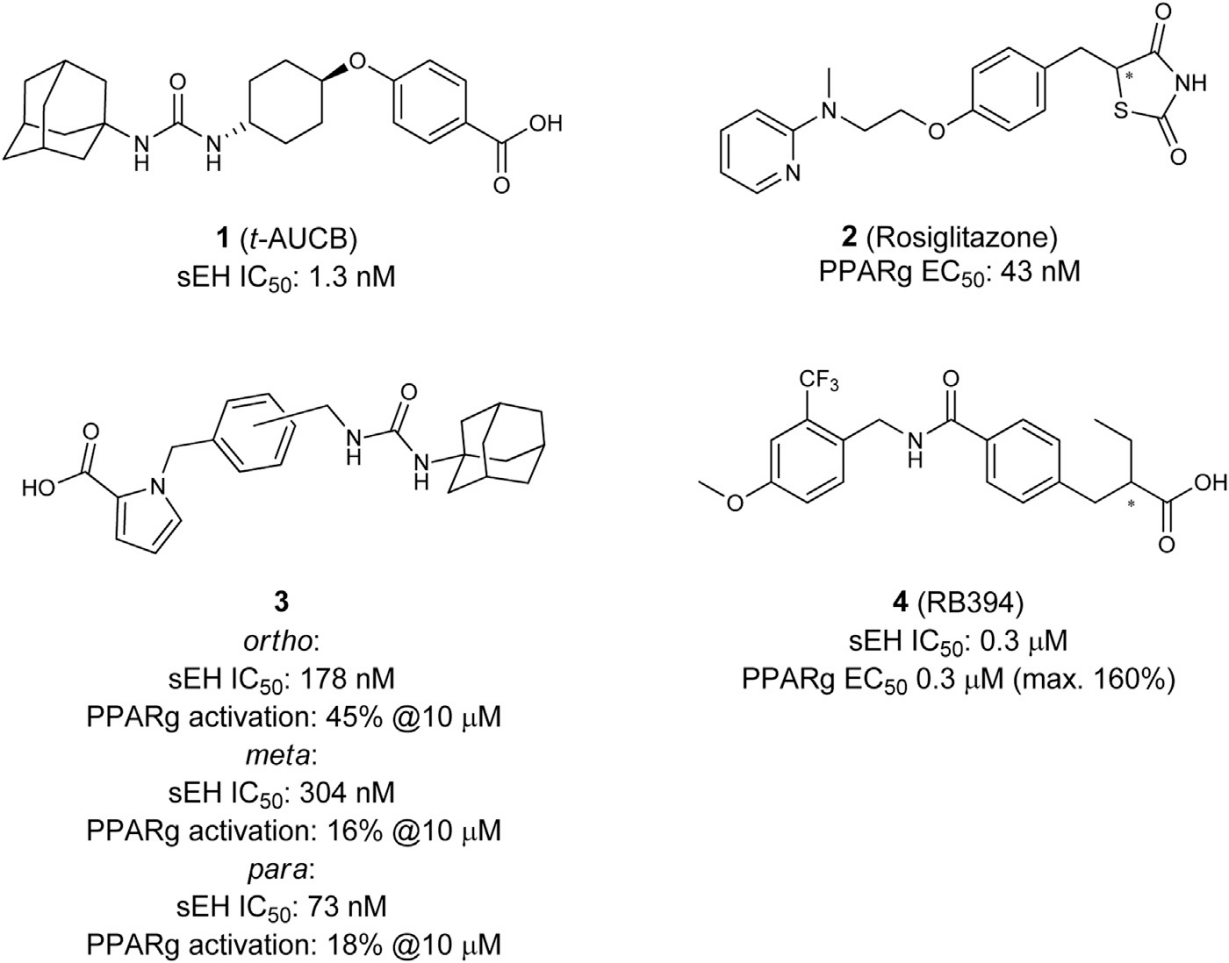
Selective sEH inhibitor t-AUCB, selective PPARγ agonist Rosiglitazone (top), and dual sEH/PPARγ modulators.

In 2012 Imig and Hammock studied the effects of the selective PPARγ agonist Rosiglitazone (**2**) (Willson et al., 2000) in combination with *t*-AUCB (**1**) (Figure 2) in order to decrease renal injury in spontaneously hypertensive obese (SHROB) rats (Imig et al., 2012). SHROB, also called Koletzky rats features a nonsense mutation in the leptin receptor. This mutation impairs the capacity of leptin to regulate food intake which causes dramatic weight gain and metabolic disorders. SHROB rats are homozygous for this mutation, whereby they show monogenetic obesity in combination with a hypertensive background. For this background several phenotype aspects are descried, such as insulin resistance, obesity, hypertension, increased plasma triglyceride levels, fatty liver and glucose intolerance (Ernsberger et al., 1999; Molinar-Toribio et al., 2014). Collectively, these features make SHROB rats an excellent and relevant model for the study of metabolic syndrome. The authors described that the combination of *t*-AUCB (**1**) and Rosiglitazone (**2**) is very effective with regards to preventing kidney damage. Both compounds lower blood pressure and blood glucose levels in a comparable manner, however the effects were not additive when administered together. Futhermore, *t*-AUCB (**1**) independently showed positive effects in preventing renal injury in contrast to the PPARγ modulator which showed positive decreasing effects on free fatty acids, plasma lipids and inflammation accompanied with an improved K_ATP_-mediated vasodilation. These findings lead the authors to the conclusion that the single application of an sEH-I or a PPARγ modulator is beneficial, but in regard to the multi disease characteristics of the MetS, the combination is even superior (Imig et al., 2012).

These studies together underline the potential for the development of a combined sEH/PPARγ modulator for the treatment of the MetS. The first attempt in that direction was done is 2002 when Buscato et al. described the first dual modulators **3** of sEH and PPARs as potential agents for the treatment of features related to the metabolic syndrome (la Buscató et al., 2012). Starting from an combinatorial approach they combined known pharmacophores of sEH (hydrophobic urea moiety) and PPAR (acidic headgroup) into one molecule. Despite other combination of dual sEH-I with PPARα- or PPARδ-modulators, they discovered the first dual sEH/PPARγ modulators, which were able to inhibit sEH with moderate potency and partially activate PPARγ.

Blöcher et al*.* discovered with *N*-benzyl benzamides a novel scaffold of orally available dual sEH/PPARγ modulators (Blöcher et al., 2016). By analyzing the structures of previously described PPARγ modulators (GSK1997132B (Sime et al., 2011), KCL (Nomura et al., 2003)), and the sEH inhibitor GSK2188931B (Kompa et al., 2013), they created a series of dual modulators based on the common *N*-benzyl benzamide substructure. The lead compound RB394 (**4**) showed submicromolar potency on both targets, good water solubility (500–375 µM) as well as metabolic stability in rat liver microsomes. These favorable properties were accompanied by an excellent *in vivo* pharmacokinetic profile in mice, qualifying RB394 (**4**) as a pharmacological tool for diabetic animal models (Blöcher et al., 2016).

The *in vivo* profile of RB394 (**4**) was further characterized by Hye Khan et al. (2018). They tested the dual modulator in rat models of the MetS and type 2 diabetes and could demonstrate its potential for targeting multiple risk factors. Two pre-clinical rat models were used in this study: SHROB- and ZSF1 (Zucker fatty/Spontaneously hypertensive heart failure F1 hybrid)-rats. In SHROB rats RB394 attenuated the development of insulin resistance, hypertension, hyperlipidemia and decreases kidney injury. In both rat models, RB394 effectively reduces kidney injury such as renal fibrosis and improves liver complications and steatosis. It could also be shown that RB394 in diabetic ZSF1 rats reduces hyperglycemia, hyperlipidemia and insulin resistance. Even though RB394 improved the insulin resistance it did not show any effect of altering the body weight, which in literature was described as interrelated (Eckel et al., 2005). The authors explained this findings thereby that the body weight gain initiated by PPARγ agonists could be caused by edema, which result of PPARγ mediated stimulation of kidney epithelial sodium channels (ENaCs) (Shah and Mudaliar, 2010). sEH-I alone did not show any effect on the body weight change, but they seem to have an effect on the ENaCs. EETs block ENaCs (Pavlov et al., 2011) and, therefore, no PPARγ activation caused body weight gain was observed, which, furthermore, highlights the potential of a dual modulator. Additionally, the preventive and reducing effects on insulin resistance and hyperinsulinaemia as well as the lipid-lowering effects in those rat models are due to the combined sEH/PPARγ pharmacophores in RB394. Finally, the dual modulator reduces MCP-1 (monocyte chemoattractant protein-1) excretion, renal immune cell infiltration and TGF-β (transforming growth factor beta) expression which in summary lead to a reduced renal inflammation. The renal protective effects were confirmed mouse unilateral ureteral obstruction (UUO) model. In this setting, RB394 outperformed a selective sEH-I and a selective PPARγ agoinist rosiglitazone, as well as their combination (Stavniichuk et al., 2020).

These findings support the hypothesis that the combination of an sEH inhibitor with a selective PPARγ agonist in one molecule could be beneficial for the treatment of type 2 diabetes, related complications, and potentially the MetS in general. The dual sEH/PPARγ modulator also possibly extends the applicability of sEH inhibitors in context of cancer promoting effects. In a hallmark publication by Panigrahy et al. epoxyeicosanoids are responsible for mild promotion of metastasis (Panigrahy et al., 2012). The effects were mainly mediated by cyclooxygenase (COX)-derived metabolites of EETs (Rand et al., 2017) and can be reversed by the application of a dual sEH/COX inhibitor (Zhang et al., 2014). Several publications demonstrated that PPARγ activation suppresses COX-2 expression (Mendez and LaPointe, 2003) and a dual sEH/PPARγ could potentially circumvents the tumor promoting effects of (COX)-derived metabolites of EETs.

### sEH and FXR

FXR (NR1H4) is a nuclear bile acid (BA) receptor (Makishima et al., 1999; Cariou and Staels, 2007). As a transcription factor, FXR binds to the response elements of its target genes either as monomer, as homodimer or as heterodimer with RXR. FXR regulates the transcription of genes that, among others, are involved in inflammation and glucose and lipid homeostasis (Carr and Reid, 2015). It is highly expressed in liver, intestine, kidney and adrenal glands, but it is also expressed in white adipose tissue and is induced during *in vitro* adipocyte differentiation (Cariou and Staels, 2007). The physiological role of FXR is liver protective, as it is an enterohepatic regulator of bile acid homeostasis (Massafra et al., 2018). The role of FXR in the context of the metabolic syndrome has been intensively reviewed in literature, underlining its potential for the treatment of the MetS (Cariou and Staels, 2007; Ma and Patti, 2014).

**FIGURE 3:**
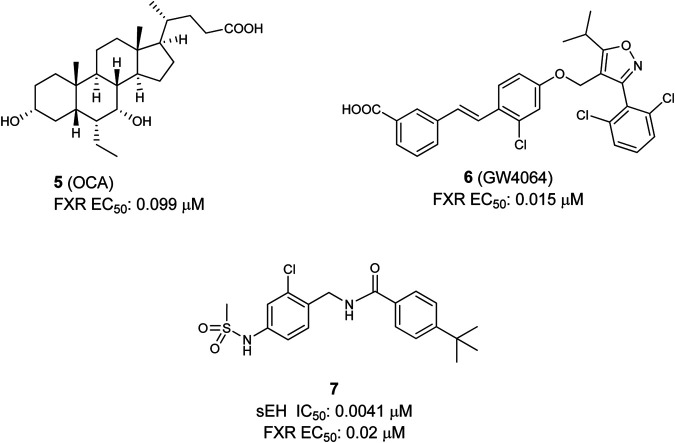
Selective FXR agonists OCA and GW4064 (top), and dual sEH/FXR modulator.

Generally two types of FXR agonists could be found, first bile acids and their semi-synthetic analogues, e.g., obeticholic acid (6α-ethyl-chenodeoxycholic acid OCA, 6-EDCA, **5**) (Pellicciari et al., 2002), and second non-steroidal compounds, such as the isoxazole derivative GW4064 (**6**) (Maloney et al., 2000) (Figure 3). These structures should be seen as representatives for both classes. Recently, OCA has been approved for the treatment of primary biliary cholangitis. Furthermore, representatives of both, bile acid analogues and non-steroidal FXR agonists like nidufexor (Chianelli et al., 2020) or tropifexor (Tully et al., 2017) are under clinical investigation for treatment of NAFLD and NASH (Mudaliar et al., 2013; Neuschwander-Tetri et al., 2015). The disease complex of NAFLD/NASH is considered to be a hepatic manifestation of the MetS (Petäjä and Yki-Järvinen, 2016). NASH is associated with numerous risk factors, such as obesity and type 2 diabetes mellitus (T2DM) (Rivera, 2008).The clinical trials with OCA have proven FXR as a target for the treatment of fatty liver disorders (Mudaliar et al., 2013; Neuschwander-Tetri et al., 2015). In addition, several studies in mice with high-fat-diet-induced fatty liver could show that a sEH inhibition, achieved either by pharmacological inhibition or by genetical deletion, has positive outcomes on the disease. These studies point out that in NAFLD/NASH the CYP epoxygenase pathway is an important regulator (Liu et al., 2012; Schuck et al., 2014; Mangels et al., 2016).

Taken together the anti-inflammatory and anti-steatotic effects of a sEH inhibitor and the positive effects after FXR activation in NASH with an FXR agonist, it shows clearly the potential of a dual sEH/FXR modulator in this disease. In this regard, the first dual modulator **7** was published in 2017 by Schmidt et al., 2017 Following a combinatorial approach, the authors merged the structures of a previously reported sEH inhibitor (Kompa et al., 2013) with an selective FXR partial agonist (Merk et al., 2014b). After several structural optimizations and a bioisosteric replacement strategy they yielded an efficacious dual modulator (see Figure 3). This dual modulator is able to partial activate FXR target gene expression and shifts the EET/DHET ratio via sEH inhibition toward the more favorable EETs. Furthermore, the compound was proven active *in vivo* in a male wild-type C57BL6/J mice-study (Schmidt et al., 2017).

The efficacy of the dual sEH/FXR modulator **7** was demonstrated in two models of NASH–the streptozotocin-induced mice and the choline-deficient high-fat diet induced mice (Takahashi et al., 2012). The dual sEH/FXR modulator **7** reduced the hepatic steatosis and fibrosis. Furthermore, it exhibited a pronounced anti-inflammatory effect, thereby demonstrating excellent efficacy in the complex setting of NASH (Hye Khan et al., 2019).

### FXR and PPARs

Besides the already mentioned positive effects of the selective FXR ligand OCA on many parameters of MetS in the FLINT trial (Neuschwander-Tetri et al., 2015), in the last decades intensive research has been done on the relation between FXR and the peroxisome proliferator-activated receptors, as reviewed in different publications (Cave et al., 2016; Lee, 2016; Preidis et al., 2017). From these reviews we want to highlight a few of the study’s which are related to the MetS.

### FXR and PPARα

PPARα (NR1C1) is highly expressed in the liver and brown fat tissue, followed by heart, kidney and small intestine (Kersten, 2014). It could be described as a master transcription factor for the metabolization of lipids in the fasted state or under conditions of energy shortage (Evans et al., 2004). The liver is the key organ in transitions between feeding and fasting states, by changing the system from energy accumulation to energy-consumption (Rui, 2014). PPARα in this regard increases the oxidation of fatty acids in the fasted state (Kersten, 2014). On the other hand, in the fed state, FXR is activated. While both nuclear receptors seem to act opposite to each other in their metabolic functions, there is clear evidence in literature that they have the same effect in suppressing lipogenesis (Watanabe et al., 2004; Savkur et al., 2005; Inagaki et al., 2007; Kersten, 2014). This could be beneficial in the case of fatty liver. McGarry suggested a vicious circle: increased steatosis levels results in insulin resistance, this in turn leads to even more steatosis all of which is triggered by an increase in lipogenesis in the insulin-resistant liver (McGarry, 1992). Moore proposed that in response to the activation of different nuclear receptors (e.g., FXR and maybe PPARα), lipogenesis is suppressed (Moore, 2012). This improves fatty liver and ameliorate insulin sensitivity and in turn further inhibits lipogenesis. In this way the vicious cycle would be reversed into a beneficial one. Because PPARα and FXR are activated in different states of the nutrient-based energy uptake and consumption, their interplay in the healthy state is well orchestrated to ensure that metabolic flux and energy balance is regulated appropriately (Preidis et al., 2017). In the unhealthy state, this energy balance is malfunctioning and, therefore, treatment with PPARα- and FXR-antagonist or perhaps a dual ligand could be beneficial if it is pharmacologically possible to target each nuclear receptor in the adequate state.

**FIGURE 4:**
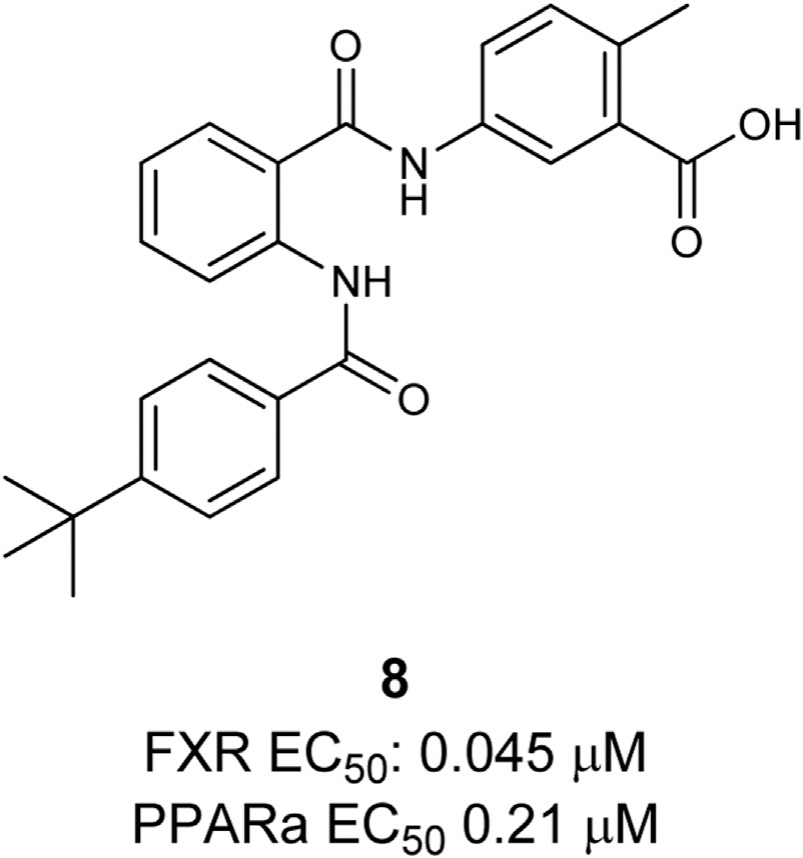
Dual FXR/PPARα agonist.

The first dual PPARα/FXR partial agonist **8** was descripted by Merk et al*.* (Figure 4). Compound **8** was found by characterizing their former published selective FXR modulating anthranilic acid derivatives on the off-targets: PPARs (α, β/δ, γ) (Merk et al., 2014a; Merk et al., 2014b). Based on these findings a novel structure–activity relationship (SAR) study was conducted in order to optimize selectivity and potency toward the PPARs, leading to the discovery of potent and selective PPAR agonists. In this way the dual PPARα/FXR agonist was developed. This partial agonist **8** is selective over other subtypes of PPAR, highly potent on both targets, and exhibits only slight toxicity (Merk et al., 2015).

All in all, the authors concluded that a dual PPARα/FXR modulator, amalgamating the positive effects of FXR and PPARα activation, could be highly beneficial and offer a possibility for the treatment of lipid associated, metabolic abnormalities. This initial study could be a good starting point for further optimization on this promising new lead structure.

### FXR and PPARγ

Besides the already descripted relation between PPARα and FXR, in literature, a connection between FXR and PPARγ has also been described. Previous studies have shown that FXR ligands are able to induce PPARα mRNA levels in human hepatic cells (Pineda Torra et al., 2003). In a rodent model of liver cirrhosis, Fiorucci et al. have shown that FXR ligands regulate PPARγ gene expression and, furthermore, that both nuclear receptors act synergistically in regulation of profibrogenetic events in human hepatic stellate cells (HSCs) (Fiorucci et al., 2005). HSCs or perisinusoidal cells are pericytes, which are involved in liver fibrosis. In addition, this study demonstrated that natural (bile acid: Chenodeoxycholic acid (CDCA)) as well as synthetic (GW4064) FXR ligands induce the expression of PPARγ in HSCs. While in liver diseases a down regulation of PPARγ is observed, the authors could show that an FXR ligand protects against that mechanism and also that the FXR ligand improves the antifibrotic activity of PPAR ligands. This is hint for a cross-talk between both nuclear receptors which could limit the activation of HSCs. Until now, no dual FXR/PPARγ ligand has been described in the literature. However, the study from Fiorucci et al*.* has shown a development of such a ligand could be worthwhile.

### ACE and DPP4

As the population numbers of T2DM continues to rise, the need for identifying pharmacological tools to conquer this disease is also increasing. A very prominent class in this context are DPP4 inhibitors, also called Gliptins (Scheen, 2015). These molecules have the potential to increase glucose uptake by inhibiting the DPP4 enzyme and preserving the action of GLP-1. GLP-1 is a peptide-hormone which belongs to the incretins and is able to liberate insulin in a dose dependent manner after oral glucose uptake. Furthermore, GLP-1 suppresses the formation of glucagon (Grigoropoulou et al., 2013). In the human body GLP-1 is rapidly degraded after formation by DPP4 which makes it challenging, but not impossible, to be targeted directly (Tomlinson et al., 2016). First GLP-1 agonist, semaglutide, was found to be very effective and entered the market (Dhillon, 2018). However, DPP4 as the GLP-1 degrading enzyme is still a valuable target to face T2DM. DPP4, also known as CD26, is an exopeptidase which cleaves proline or alanine dipeptides from the *N*-terminus of proteins and peptides. This cleavage leads to the building of new biological active peptides or inactivates those proteins/peptides. DPP4 is expressed in various tissues and can either be found membrane bound or as a soluble circulating form (sDDP4) liberated from the plasma membrane without its intracellular tail and transmembrane regions (Durinx et al., 2000). Several gliptins have already come onto the market for the treatment of T2DM. This class of inhibitors exhibit different side effects such as skin reactions or gastrointestinal problems (Charbonnel et al., 2006; Nelson et al., 2014; Scheen, 2018). This underlines as well the potential of inhibiting DPP4, but also the need for new and safer inhibitors for this enzyme.

While DPP4 inhibitors are effective against hyperglyceamia, for the treatment of hypertension ACE (angiotensin converting enzyme) inhibitors have been shown to be efficient and safe form of treatment. ACE inhibitors are a class of antihypertensive drugs, which is primarily used for the treatment of cardiovascular (e.g., congestive heart failure (CHF)), or renal diseases (Regulski et al., 2015). Prominent examples are the substances captopril, enalapril or benazepril. This class of molecules acts through the inhibition of the angiotensin converting enzyme and through this mechanism are able to decrease activity of the renin-angiotensin-aldosterone system (RA(A)S). RAS controls the arterial blood pressure, electrolyte balance, and cardiovascular, adrenal and renal functions (Ferrario and Strawn, 2006; Regulski et al., 2015). To do so, the proteolytic enzyme renin is released in the kidney. There, it tranforms its substrate angiotensinogen into angiotensin I (Ang I). AngI is then further cleaved bye ACE to the physiologically active angiotensin II (Ang II) (Pacurari et al., 2014). Renin is released for example in response to low blood pressure, hyponatremia, or potassium depletion (Regulski et al., 2015). Therefore, the inhibition of the conversion of Ang I to Ang II leads very general to a decrease in blood pressure and vascular tone. Moreover, it has been shown that ACE inhibitors are able to reduce the progress of diabetic nephropathy and provide renovascular protection (Feng et al., 2019).

Wang et al. reported in 2012 the discovery of an egg protein hydrolysate, which shows DDP4- and ACE-inhibitory activity (Wang et al., 2012). Following an *in silico* approach to identify a protein that is able to modulate relevant MetS-targets, the authors analyzed protein digests and calculated their bioactivity, leading to the finding of the egg protein lysozyme NWT-03. After digestion with alcalase, this protein has the potential to act as a protein precursor for ACE-inhibitory peptides. By confirming the ACE-inhibition activity in a subsequent *in vitro* assay (IC_50_ = 0.07 mg/ml), their results also revealed that NWT-03 could be able to inhibit DPP4 (IC_50_ = 0.9 mg/ml). With this dual acting peptide, the authors wanted to address the question whether it could also beneficially affect T2DM developed parameters of renovascular damage. For that purpose, renal damage and vascular dysfunction was reviewed in an animal study in Zucker diabetic fatty (ZDF) rats, where NWT-03 was supplemented in drinking water (1 g/kg/day) over a period of 15 weeks. The results were compared to a parallel ZDF rat group who were treated with the DPP4-Inhibitor vildagliptin (15 weeks, orally in drinking water, 3 mg/kg/day), which functions as a positive control for the effect of DDP4 inhibition. With this rat model, the authors showed that NWT-03 attenuates the development of renal damage. Furthermore, it has a preventing effect on aortic endothelial dysfuntion. The peptide is able to reduce albuminuria and focal glomerulosclerosis (FGS) by about 50% and moreover, is able to improve impaired endothelium dependent relaxation (EDR) response to acetylcholine (AcH). The results of the vildagliptin treated control-group showed that this selective inhibitor has a positive effect on cytokines related to inflammation and glomerulosclerosis, but these effects are weaker when compared to NWT-03. Because of this, the authors concluded that the more favorable effects of NWT-03 over vildagliptin are due to its ability to act dual on ACE and DPP4 (Wang et al., 2012).

**FIGURE 5:**
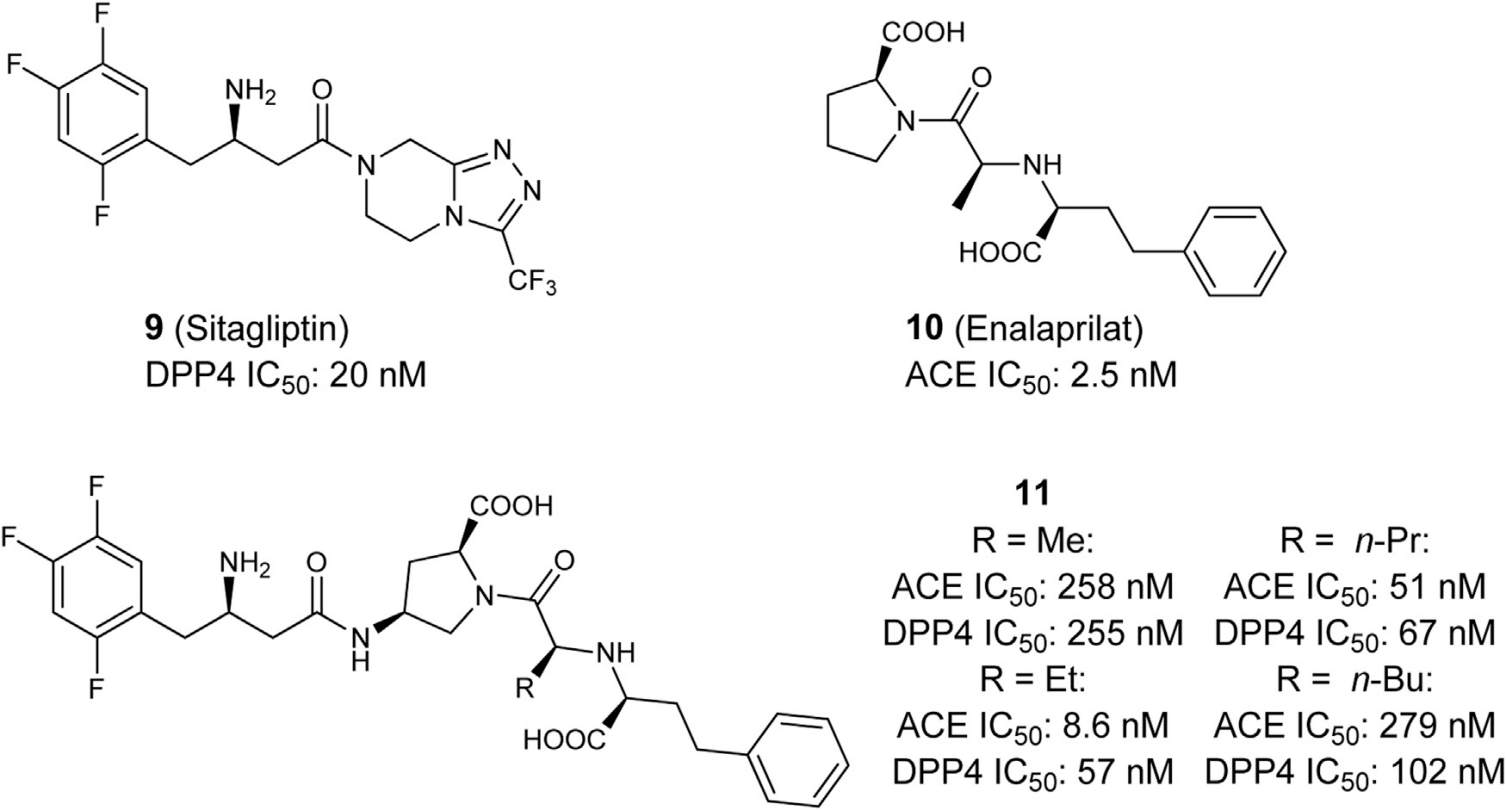
Selective DPP4 inhibitor Sitagliptin, selective ACE inhibitor Enalaprilat, and dual ACE/DPP4 inhibitor.

In 2017 Sattigeri et al*.* presented the first approach toward a small molecule-based dual ACE/DPP4 inhibitor for the treatment of MetS (Figure 5) (Sattigeri et al., 2017). By analyzing ligand co-crystal structures of the DDP4-inhibitor sitagliptin (**9**) and the ACE inhibitor enalaprilat (**10**), critical potency-relevant interactions for both enzymes were identified. As a result of this approach the authors successfully merged the most relevant functional groups of enalaprilat, the active metabolite of enalapril and sitagliptin, together and received a new lead structure **11**. Four derivatives with different terminal alkyl-residues were successfully characterized via molecular docking. The new ligands fit into the binding pockets very well and shows similar binding modes/molecular interactions compared to those already reported in literature. *In vitro* characterization of the molecules was performed in rat- (Wistar), mouse- (*ob*/*ob*) and human-plasma as an enzyme source. The results showed clearly that all compounds **11** are able to inhibit both enzymes, especially in human subjects with good potency, but the compounds also partially showed a very large inter-species shift. Nearly all compounds showed an excellent selectivity profiles against DASH- (dipeptidyl peptidase-IV activity and/or structure homologues)-enzymes (DPP2, DPP8, DPP9) and non-DASH-enzymes (post-proline cleaving enzyme (PPCE), neutral endopeptidase (NEP), aminopeptidase P (APP) and aminopeptidase N (APN). Furthermore, the compounds **11** were stable in rat, mouse, dog and human liver microsomes. The plasma exposure profiles (Wistar rats following oral dosing, 30 mpk) are partially good. Overall the authors have successfully presented the first approaches toward a dual ACE/DPP4-inhibitor which exhibits good and balanced potency on both targets and an advantageous *in vitro* profiles (Sattigeri et al., 2017).

### PPARγ and AT1

The discovery of dual PPARγ agonists which additionally antagonize AT1 receptors can be considered as a design-in approach. AT1 receptors are expressed in various tissues, most importantly kidney, heart, lung, and vessel walls (Kakar et al., 1992; Gasc et al., 1994). AT1 receptor responses to the binding of Angiotensin II and its activation leads to vasoconstriction, increased sodium reabsorption, and aldosterone levels (Schmieder, 2005). Selective antagonists of the AT1 receptor are also referred to as sartans. The pharmacological properties of AT1 receptor antagonists have been excessively reviewed previously (Timmermans, 1999). Since the discovery of first approved non-peptidic AT1 receptor antagonist losartan (Duncia et al., 1990), various sartans have been approved as first-line therapy for treatment of hypertension (Naik et al., 2010).

**FIGURE 6:**
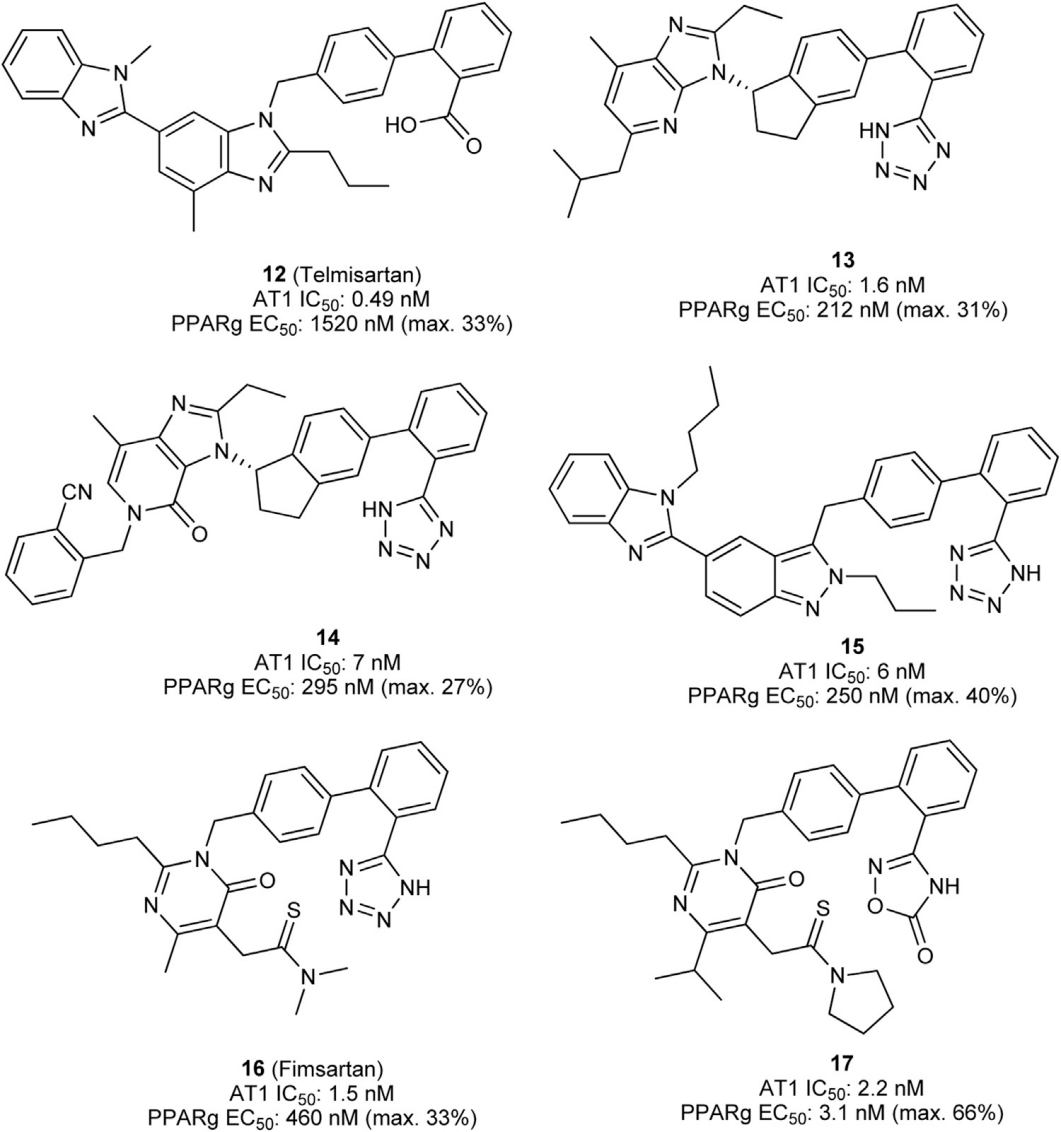
Telmisartan and designed dual PPARγ/AT1 ligands.

The first dual PPARγ/AT1 receptor ligand is telmisartan (**12**, Figure 6), an antihypertensive drug discovered by Boehringer Ingelheim (Ries et al., 1993). Initially, telmisartan was considered to be a selective antihypertensive drug exhibiting sub-nanomolar IC_50_ value toward the AT1 receptor. Benson et al. discovered PPARγ modulating activity of telmisartan (Benson et al., 2004). Various clinical trials were performed to translate and confirm the beneficial effects of telmisartan compared to other AT1 receptor antagonists on glucose and lipid metabolism, however, these results were inconsistent (Derosa et al., 2004; Vitale et al., 2005; Hamada et al., 2014; Naruse et al., 2019). Meta-analysis of randomized controlled trials confirmed superiority of telmisartan in improvement of insulin resistance, reduction of fasting blood glucose and blood insulin levels (Wang et al., 2018). Telmisartan modulates PPARγ at low micromolar concentrations, the difference in effective concentration for pharmacodynamic modulation of PPARγ and AT1 receptor is quite high. Therefore, research has been conducted to design-in more potent modulation of PPARγ while preserving AT1 receptor antagonistic activity of telmisartan.

Casimiro-Garcia et al. presented two series of compounds which were derived from the telmisartan, developed at Pfizer (Casimiro-Garcia et al., 2011; Casimiro-Garcia et al., 2013). The lead compound for optimization was identified by screening AT1 receptor antagonists from previous lead optimization campaign. Subsequent multidimensional optimization led to identification of compound **13**. **13** antagonized AT1 receptor with an IC_50_ of 1.6 nM and modulated PPARγ activity with an EC_50_ of 212 nM (Casimiro-Garcia et al., 2011). Similar results were obtained with compound **14** which exhibits a similar scaffold (Casimiro-Garcia et al., 2013). Very good pharmacokinetic profile allowed for testing in two distinct animal models. **14** effectively demonstrated pronounced and dose-dependent blood pressure lowering in spontaneously hypertensive rats (SHR). At the same time **14** lowered the blood glucose levels in male ZDF rats, which was far more pronounced compared to telmisartan (Casimiro-Garcia et al., 2011).

Lamotte et al. optimized the PPARγ modulation by switching the benzimidazole central core to indole, pyrazolopyridine, and indazole. The most promising indazole compound **15** exhibited an IC_50_ of 6 nM toward AT1 receptor and modulated PPARγ activity with an EC_50_ of 250 nM. Very good exposure and bioavailability in rats allowed for testing in SHR where **15** significantly lowered systolic and diastolic blood pressure. In Zucker *fa*/*fa* rats, **15** reduced the plasma insulin and plasma triglycerides. Interestingly, no body weight gain, a typical side effect of PPARγ full agonists, was observed in comparison to vehicle control (Lamotte et al., 2014).

In a more recent study by Choung et al., fimasartan (**16**) (Kim et al., 2012), an AT1 receptor antagonist approved in South Korea, was used as a starting point for optimization of PPARγ modulatory activity (Choung et al., 2018). The resulting dual modulator **17** reached higher activation efficacy compared to the lead structure and exhibited antihypertensive activity *in vivo*.

### PPARγ and GK

Aside from the combination of an antihypertensive and antidiabetic activity in a designed multitarget ligand (DML), enhancement of antidiabetic activity by means of addressing two targets seems to be a valuable strategy. An example of a rational strategy to a synergistic antihyperglycaemic DMLs is the combined activation of PPARγ and Glucokinase (GK). GK catalyzes the phosphorylation of glucose. The product of GK-catalyzed reaction, Glucose-6-phospate, can subsequently enter the glycolysis or being incorporated into glycogen. Thus, activation of GK efficiently promotes the storage of glucose from blood in hepatocytes. Furthermore, GK acts as a glucose sensor in pancreatic islet cells and promotes insulin secretion (Toulis et al., 2020). A DML which is able to activate GK and PPARγ simultaneously has the potential to synergistically decrease the glucose levels, promote insulin secretion and sensitize muscle cells to insulin response.

**FIGURE 7:**
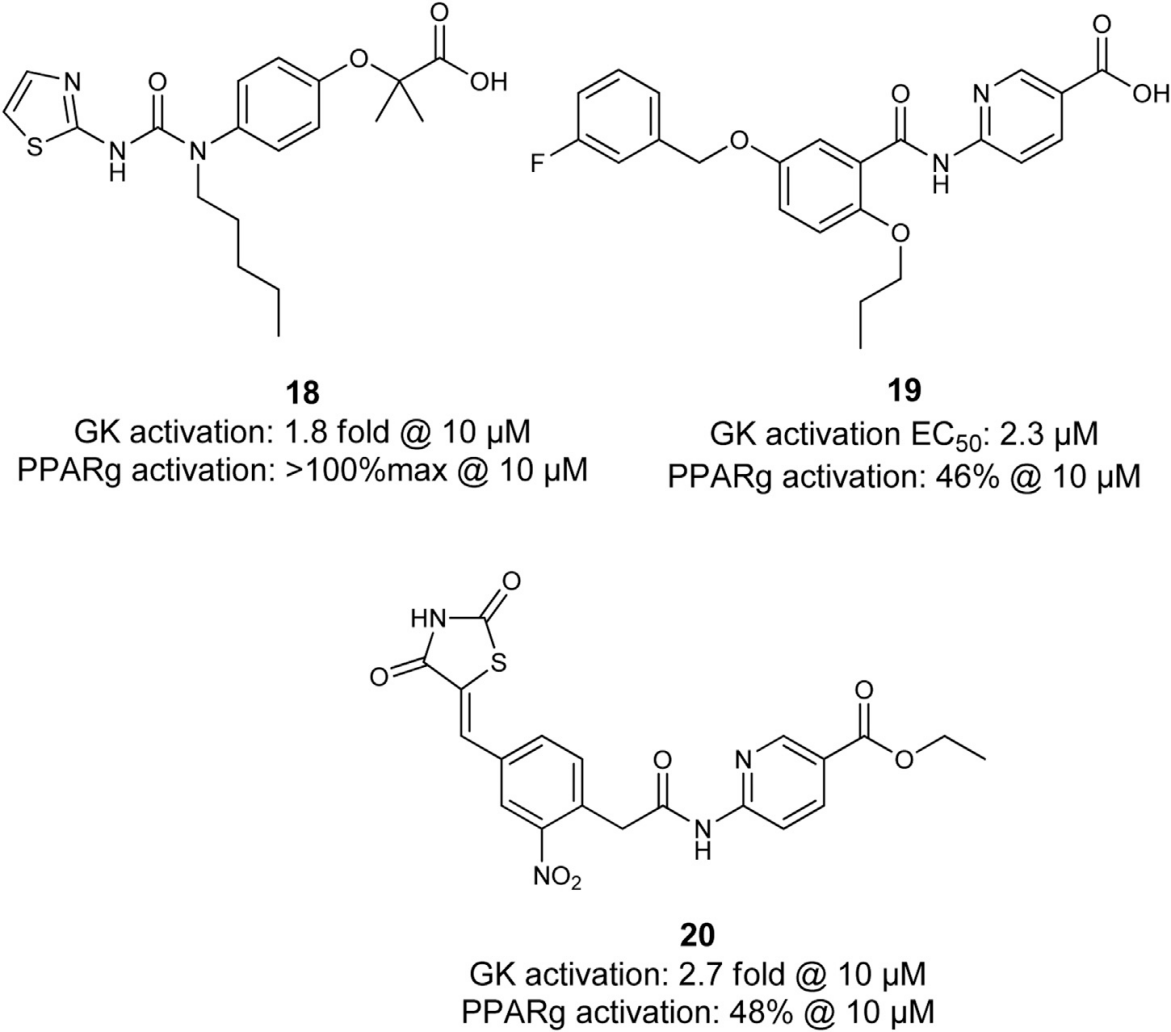
Dual PPARγ/GK modulators.

Lu et al. coupled diverse GK activator pharmacophores into thiazolidinediones which are well-known as PPARγ agonists. Although several compounds, including **20**, were shown to exhibit dual activity of both, GK and PPARγ, further evaluation is not reported (Lu et al., 2014). Li et al. linked a benzthiazole- and thiazole-2-urea moiety, which is described as a pharmacophore for potent GK activation to a fibrate pharmacophore (Figure 7). The subsequent investigation of the length of the tertiary urea alkyl substituent and substitution patterns of the heterocycle led to discovery of **18**. Compound **18** activated GK and PPARγ in low micromolar range. However, the *in vivo* glucose lowering effects observed during the fasting blood glucose tests in ICR mice upon exposure were very moderate which was caused by very rapid clearance of **18** from the blood (Li et al., 2014).

Compound **19** (SHP289–03) was found by screening for dual GK/PPARγ activators and is a derivative of the GK activator GKA 22 discovered by AstraZeneca. It partially activates PPARγ and exhibits 1.7fold activation of GK at 50 µM. Interestingly, **19** acts as a partial PPARγ agonist and does not promote adipocyte differentiation while it leads to mRNA increase of PPARγ dependent gene Adrp and aP2. In contrast to compound **18**, compound **19** (SHP289–03) exhibits much better pharmacokinetic properties in rats. In type 2 diabetic KKA^y^ mice, **19** leads to a pronounced and dose-dependent improvement of hyperglycemia and insulin secretion. Surprisingly, lipid parameters and body weight were improved in animals treated with **19** for 25 days at doses of 25 mg/kg and 50 mg/kg, demonstrating the potential of dual GK/PPARγ activators as drugs for treatment of MetS (Lei et al., 2015).

### DPP4 and GPR119

As described above, treatment with DPP4 inhibitors leads to an increase of GLP-1 in plasma and subsequent secretion of insulin in response to elevated blood glucose levels. An increased glucose-dependent insulin secretion in pancreatic β-cells as well as an increased release of GLP-1 in intestinal L-cells can also be achieved by the activation of GPR119 (Yang et al., 2018), a G-protein coupled receptor (GP(C)R), which is mainly expressed in the small intestine and in the pancreas (Odori et al., 2013). Therefore, GPR119 agonists could improve glucose homeostasis in patients with T2DM. The search for endogenous ligands of GPR119 yielded two main classes, amide derivatives of fatty acids, such as oleoylethanolamides (OEA), and phospholipids, such as oleyol-lysophosphatidylcholine (Hansen et al., 2012). In addition, a large number of synthetic compounds have already been identified which can activate GPR119 effectively and selectively (Ritter et al., 2016). Preclinical and clinical studies with various compounds have shown the potential for the treatment of T2DM, but there is still a long way to go before a candidate is approved. Due to the complementary mechanisms of action of DPP4 inhibitors and GPR119 agonists, numerous studies investigated and confirmed the synergistic effect of this target combination (Ansarullah et al., 2013).

The first dual DPP4/GPR119 modulator was introduced in 2016 by Li et al. (2016) They analyzed the most important pharmacophore properties of the DPP4 inhibitor linagliptin (xanthine scaffold) and the three GPR119 agonists AR231453, APD597, and PSN632408 (4-piperidine moiety) and developed a lead structure in a pharmacophore merging approach. A systematic optimization led to compound **21**. This dual modulator is able to activate GPR119 (EC_50_: 0.95 μM, 81.5% activity of AR231453 @1 μM) and inhibit DPP4 (IC_50_: 0.22 μM) *in vitro*. **21** also exhibits favorable selectivity against DPP8/9 (36.8 % inhibition @10 μM) as well as an acceptable *in vitro* pharmacological profile.

Optimization of **21** by Huan et al. led to compound **22** (Huan et al., 2017). **22** is able to inhibit DPP4 *ex vivo* (IC_50_: 0.066 μM) as well as *in vivo* (ICR mice, 30 mg/kg, 50% DPP4 inhibition for about 4h) and maintains selectivity against DPP8/9. GPR119 is activated *in vitro* by **22** (EC_50_: 0.03 μM, 73.6%activity of APD597 @10 μM). It is also selective via other GPCRs such as GPR40, GLP1R and GIPR. Further ex and *in vivo* experiments support the authors' statement that the dual DPP4/GPR119 modulator **22** could be a very promising candidate for the treatment of T2DM. In 2020 the same group published the SAR that led to the identification of **22** (Li et al., 2020). An improvement was acchived by using the hydrochloride salt of **22** which displays an improved bioavailability and blood sugar lowering effect in ICR mice as well as a favorable PK/PD. A mini-AMES test was negative and also the preliminary acute toxicity in mice shows an LD_50_ of more than 1.5 g/kg in first tests. The moderate inhibition of the hERG channel (IC_50_: 4.9 μM), which according to the authors’ hypothesis is due to the high lipophilicity of the compound, represents a challenge to be solved in the future.

In 2020, Fang et al. presented another dual modulator, the tetrahydropyrimidine derivative **23** (Fang et al., 2020). Based on the previously developed GPR119 agonists (Fang et al., 2019), the dual modulator was designed via pharmacophore combination and scaffold hopping. A central tetrahydropyrimidine nucleus was combined with a substituted aniline and seven different DPP4 inhibitors from literature, which led to the identification of **23**. The compound displays inhibitory activity against DPP4 (74.5% @10 μM) and is also a highly potent GPR119 agonist (EC_50_: 0.0087 μM, in a cell-based cAMP assay, 85.4 %max; compared to max. effect of GSK1292263). The calculated clogP of 2.1 indicates a suitable lipophilicity. In an oral glucose tolerance test in C57BL/6N mice **23** also showed a stronger hypoglycemic effect than the DPP4 inhibitor vildagliptin.

### DPP4 and MCH-1R

As already mentioned, obesity is one of the central risk factors of MetS. One of the many approaches to treat obesity is appetite control (Sargent and Moore, 2009). A receptor that could play an important role in this context in the future is the melanin concentrating hormone receptor 1 (MCH-1R/ GPR24/ SLC-1). This GPCR is activated by the melanin-concentrating hormone (MCH), a cyclic neuropeptide with 19 amino acids, which is synthesized in mammals primarily by neurons in the lateral hypothalamus (LH) and the zona incerta of the subthalamus. The important role MCH has with regards to food intake and energy balance has been investigated and confirmed in numerous animal studies (Luthin, 2007; Mihalic et al., 2011). In addition to MCH-1R, there is also a second MCH receptor, MCH-2R, whose physiological role in humans has not been elucidated yet (Omran, 2017). A number of MCH-1R antagonists have already been described in the literature (Johansson and Löfberg, 2015) and it could be shown early on that they suppress MCH-induced food intake and thus lead to a reduction in weight gain (Borowsky et al., 2002; Takekawa et al., 2002). A problem in the development of this compound class is the correlation of the structural as well as physico-chemical requirements for MCH-1R potency and hERG channel inhibition resulting in an increased cardiovascular risk (Högberg et al., 2012). Up to now, only a few candidates were investigated in clinical trials. For various reasons, however, in no case has phase 1 been exceeded and the concept of weight loss mediated by MCH-1R inhibition has thus not been confirmed in humans yet (Omran, 2017). Although the development of selective MCH-1R antagonists is associated with a number of challenges, the combination with a DPP4 inhibitor could be an attractive combination resulting in an oral anti-diabetic agent which would also contribute to reduction of body weight.

Gattrell et al. presented the first approach to the development of a dual DPP4/MCH-1R modulator (Gattrell et al., 2012). Based on ligand data from the chemical literature and patents they identified molecules that share a common structural motif and developed a lead structure. Subsequently, they identified further MCH-1R ligands with a similar binding mode via molecular docking, fragmented them and filtered them according to predetermined physicochemical properties. The resulting initial set of analogues was tested and, after further minor modifications, compound **24** was obtained. Analysis of the MCH-1R antagonism (IC_50_: 0.44 μM) and DPP4 inhibition (IC_50_: 0.35 μM) showed that **24** is almost equipotent on both targets and also selective against DDP8/9. The hERG inhibition data of two analogues of **24** also suggest that the presented chemotype is a good starting point for further lead structure optimization.

## Conclusion

In conclusion, the summarized studies indicate the potential of facing a multifactorial disease by combining two selective inhibitors into one molecule with dual modulator activity. There are multiple reasons for introducing multi-target activity in compounds for treatment of MetS (Table 1). First, avoidance of polypharmaceutical treatment can be potentially achieved by combining anti-hypertensional and anti-diabetic activity as it was shown for AT1/PPARγ, sEH/PPARγ, or ACE/DPP4 ligands. The *in vivo* evaluation of most advanced compounds demonstrates great potential in efficacy; however, clinical trials are lacking. Second, enhancement of efficacy against particular MetS associated morbidities can be achieved by combining targets such as sEH/FXR or GK/PPARγ. This approach also holds great potential to reduce polypharmacological treatment due to the fact that these diseases are managed with multiple drugs at the moment. Medicinal chemistry demonstrated the capability to deliver potent multitarget modulators with favorable pharmacokinetic properties. Furthermore, innovative animal models of MetS are able to reflect the whole spectrum of symptoms, thereby are able to reflect the whole spectrum of symptoms such that the full potential of multitarget ligands can be evaluated. A future perspective for these compounds should be evaluated in clinical trials to demonstrate the full potential of multitarget drugs in patients.

**TABLE 1:** Beneficial effects of combinations of pharmacological targets.

*Target combination*	sEH/PPARγ	DPP4/MCH-1R	DPP4/GPR119	DPP4/ACE	
*Positive implications for MetS*	Antidiabetic, cardioprotective, renoprotective, blood pressure-lowering	Antidiabetic, weight loss	Antidiabetic, glucose homeostasis	Antidiabetic, treating hyperglycemia and hypertension	
*Target combination*	sEH/FXR	FXR/PPARα	FXR/PPARγ	PPARγ/AT1	PPARγ/GK
*Positive implications for MetS*	Anti-inflammatory, anti-steatotic	Suppressing lipogenesis	Antifibrotic, liver protective	Antihypertensive, antidiabetic	Improves insulin resistance, reducing blood glucose levels

## Glossary

AA: arachidonic acid
ACE: angiotensin converting enzyme
AcH: acetylcholine
AMP: adenosine monophosphate
Ang I/ II: angiotensin I/ II
APP: aminopeptidase P
APN: aminopeptidase N
AT1: angiotensin II receptor subtype 1
BA: bile acid
cAMP: cyclic adenosine monophosphate
CB1: cannabinoid type 1
CDCA: Chenodeoxycholic acid
CHF: congestive heart failure
COX: cyclooxygenase
CYP: cytochrome P450
DASH: dipeptidyl peptidase-IV activity and/or structure homologues
DHETs: dihydroxyepoxyeicosatrienoic acids
DML: designed multitarget ligand
DPP4: dipeptidyl peptidase-4
EDR: endothelium dependent relaxation
EETs: epoxyeicosatrienoic acids
ENaCs: epithelial sodium channels
EPC: epithelial progenitor cells
FGS: focal glomerulosclerosis
FLINT: Farnesoid X nuclear receptor ligand obeticholic acid for non-cirrhotic, non-alcoholic steatohepatitis
FXR: farnesoid X receptor
GK: Glucokinase
GLP-1: glucagon-like peptide-1
GP(C)R: G-protein coupled receptor
HbA1c: hemoglobin A1c
hERG: human ether-a-go-go-related gene
HETE: hydroxyeicosatrienoic acid
HIF-1α: Hypoxia-inducible factor 1-alpha
HSCs: human hepatic stellate cells
IL-6: interleukin-6
HDL: high-density lipoprotein
HMG-CoA 3: hydroxy-3-methyl-glutaryl-coenzyme A
LDL: low-density lipoprotein
LH: lateral hypothalamus
MCH: melanin-concentrating hormone
MCH-1R: melanin concentrating hormone receptor 1
MCP-1: monocyte chemoattractant protein-1
MetS: Metabolic syndrome
NAFLD: non-alcoholic fatty liver disease
NASH: non-alcoholic steatohepatitis
NEP: neutral endopeptidase
OCA: obeticholic acid
OEA: oleoylethanolamides
PPARs: proliferator-activated receptors
PPCE: post-proline cleaving enzyme
RA(A)S: renin-angiotensin-aldosterone system
RXR: retionoid-X-receptor
SAR: structure–activity relationship
sDDP4: soluble dipeptidyl peptidase-4
sEH: soluble epoxide hydrolase
sEH-I: soluble epoxide hydrolase-inhibitor
SGLT-2: sodium-glucose cotransporter-2
SHR: spontaneously hypertensive rats
SHROB rat: spontaneously hypertensive obese rat
T2DM: type 2 diabetes mellitus
TGF-β: transforming growth factor beta
TNF-α: tumor necrosis factor-α
TZD: thiazolidinedione
UUO: unilateral ureteral obstruction
VEGF: vascular endothelial growth factor
ZDF: ratzucker diabetic fatty rat
ZSF1: ratzucker fatty/spontaneously hypertensive heart failure F1 hybrid rat
